# A Novel In Vitro Primary Human Alveolar Model (AlveolAir™) for H1N1 and SARS-CoV-2 Infection and Antiviral Screening

**DOI:** 10.3390/microorganisms13030572

**Published:** 2025-03-03

**Authors:** Cindia Ferreira Lopes, Emilie Laurent, Mireille Caul-Futy, Julia Dubois, Chloé Mialon, Caroline Chojnacki, Edouard Sage, Bernadett Boda, Song Huang, Manuel Rosa-Calatrava, Samuel Constant

**Affiliations:** 1 Epithelix,1228 Geneva, Switzerland; cindia.ferreira@epithelix.com (C.F.L.); mireille.caulfuty@epithelix.com (M.C.-F.); caroline.chojnacki@epithelix.com (C.C.); bernadett.boda@epithelix.com (B.B.); song.huang@epithelix.com (S.H.); 2 CIRI, Centre International de Recherche en Infectiologie, Team VirPath, Inserm, Université Claude Bernard Lyon 1, CNRS, UMR5308, ENS de Lyon, 69007 Lyon, France; emilie.laurent@univ-lyon1.fr (E.L.); julia.dubois@univ-lyon1.fr (J.D.); chloe.mialon@univ-lyon1.fr (C.M.); manuel.rosa-calatrava@univ-lyon1.fr (M.R.-C.); 3 Virnext, Faculté de Médecine RTH Laennec, Université Claude Bernard Lyon 1, 69008 Lyon, France; 4 International Research Laboratory RespiVir France-Canada, Centre de Recherche en Infectiologie, Faculté de Médecine RTH Laennec Université Claude Bernard Lyon 1, INSERM, CNRS, ENS de Lyon, 69008 Lyon, France; 5 Hôpital Foch, 92150 Suresnes, France; e.sage@hopital-foch.com; 6 Centre de Recherche en Infectiologie, Centre Hospitalier Universitaire de Québec, Université Laval, Québec, QC G1V 4G2, Canada

**Keywords:** respiratory viruses, H1N1, SARS-CoV-2, alveolus, barrier function, in vitro, air–liquid interface culture, type I and II primary pneumocytes

## Abstract

Lower respiratory infections, mostly caused by viral or bacterial pathogens, remain a leading global cause of mortality. The differences between animal models and humans contribute to inefficiencies in drug development, highlighting the need for more relevant and predictive, non-animal models. In this context, AlveolAir™, a fully primary in vitro 3D human alveolar model, was characterized and demonstrated the sustained presence of alveolar type I (ATI) and type II (ATII) cells. This model exhibited a functional barrier over a 30-day period, evidenced by high transepithelial electrical resistance (TEER). These findings were further validated by tight junctions’ confocal microscopy and low permeability to Lucifer yellow, confirming AlveolAir™ as robust platform for drug transport assays. Additionally, successful infections with H1N1 and SARS-CoV-2 viruses were achieved, and antiviral treatments with Baloxavir and Remdesivir, respectively, effectively reduced viral replication. Interestingly, both viruses infected only the epithelial layer without replicating in endothelial cells. These findings indicate AlveolAir™ as a relevant model for assessing the toxicity and permeability of xenobiotics and evaluating the efficacy of novel antiviral therapies.

## 1. Introduction

Animal models have historically served as a foundational tool in life sciences research, significantly contributing to the understanding of fundamental biological mechanisms. They have been a requisite component of drug development, serving as a preliminary step prior to clinical trials in humans. However, the translational success of findings from animal models to human clinical trials remains limited. Only approximately 10% of studies that transition from animal models to randomized human trials receive approval for patient use [1]. This low success rate is primarily attributed to interspecies differences at various biological levels. For example, mouse models were demonstrated to be inadequate in accurately replicating human inflammatory diseases, as evidenced by the even lower success rate of clinical trials in the sector of inflammation [2]. Similarly, poor translation between rats and humans is observed in the field of respiratory infections and diseases. These data highlight the importance of human cell-based systems for more accurate toxicological assessments [3]. 

In this context, the Tox21 Consortium, a collaborative U.S. federal research initiative, has sought to enhance efficiency in evaluating the potential health effects of thousands of chemicals. By prioritizing the advancement of alternative, non-animal testing methodologies, the consortium also contributes to enhancing drug development [4].

Among such efforts, understanding the human respiratory system is critical, as it plays a pivotal role in drug delivery and toxicological assessments. In humans, the lung parenchyma, where gas exchange occurs, accounts for approximately 90% of the total lung volume, while the remaining 10% consists mainly of conducting airways. Alveoli within the parenchyma are connected to the distal airways, forming functional clusters known as acini. Each acinus begins with a transitional bronchiole and includes alveolar ducts and respiratory bronchioles, both of which contribute to gas exchange. The inter-alveolar septum is critical for gas exchange, separating the air-filled alveolar space from the blood-filled capillary lumen. To optimize oxygen and carbon dioxide diffusion, the septum provides a large surface area (around 140 m^2^ in humans) and a thin diffusion barrier (around 2 µm). Structurally, it comprises two cell layers: an alveolar epithelium facing the airspace and a capillary endothelium facing the blood. Between them lies an interstitial space of variable thickness. The alveolar epithelium includes two specialized cell types: Alveolar Type I (ATI) cells, which are large, flat cells that cover around 95% of the alveolar surface and facilitate efficient gas exchange, and Alveolar Type II (ATII) cells, which are cuboidal cells containing surfactant-storing lamellar bodies. These cells synthesize, secrete, and recycle pulmonary surfactant, composed of 90% lipids and 10% proteins [5]. Surfactant enhances alveolar stability by reducing surface tension at the air–liquid interface [6]. ATII cells also serve as progenitors, capable of self-renewal and differentiating into ATI cells following epithelial injury [5].

Beyond structural and metabolic roles, ATII cells are essential for immune defense. They regulate alveolar inflammation in response to injury and exhibit innate immune responses, such as upregulating interferon-β and interferon-λ mRNA and secreting proinflammatory cytokines and chemokines during SARS-CoV-2 infection [7,8]. The COVID-19 pandemic and seasonal influenza and respiratory syncytial virus epidemics underscored the significant threat posed by respiratory viruses to human health. It highlighted the urgent need for reliable in vitro models using primary human cells. Such models could accelerate the discovery of effective drugs or the repurposing of existing therapies. During the last decades, a variety of human cell-based in vitro models have been developed. Various settings and devices have been used to model human alveoli mostly as precise cut lung slices, air–liquid interface cultures, spheroids, organoids, and lung-on-a-chip [9,10,11,12,13,14,15,16,17,18,19,20,21,22,23,24,25]. Significant efforts have been dedicated to develop these models, which continue to play a crucial role in advancing our understanding of respiratory biology and disease [26].

Despite these advancements, several limitations remain, varying by type of model. Common challenges include restricted usability over time, lack of tissue tightness, non-physiological exposure to stimuli, difficulties in model handling, low throughput, and the rapid loss of ATIIs. To address these challenges, the objective of this study was to develop AlveolAir™, an in vitro alveolar co-culture system comprising primary alveolar epithelial and endothelial cells. This model aims to overcome existing challenges and provide a reliable model for studying viral infections. First, we characterized ATI and ATII cells in this alveolar model cultivated at the air–liquid interface (ALI) and their barrier function up to 30 days was evaluated. Moreover, AlveolAir™ was successfully infected by H1N1, which resulted in a strong inflammatory response as well as strong impact on tissue integrity. The addition of antiviral Baloxavir reduced viral replication as well as inflammatory cytokines production compared to non-treated infected tissues. Infection of AlveolAir™ by the Delta variant of SARS-CoV-2 induced interferon genes upregulation and had no impact on tissue integrity. The addition of antiviral Remdesivir completely prevented Delta replication, leading to a non-infected phenotype. These results evidence a useful ready-to-use primary alveolar model for antiviral drug discovery.

## 2. Materials and Methods

### 2.1. Cells Isolation and Culture

Epithelial and endothelial cells were isolated from the lungs obtained from patients undergoing lung lobectomy. The donors of the lungs, or their relatives, have given, read, and signed an informed consent form, which was kept in the tissue collection centers. The study was conducted according to the declaration of Helsinki on biomedical research (Hong Kong amendment, 2013) and received approval from the local ethics commission. The epithelial and endothelial cells were isolated from the distal lungs by enzymatic digestion. After amplification, the cells were seeded on the microporous membrane of Transwell^®^ inserts (24-well format, Oxyphen, Lottstetten, Germany) and grown in a defined alveolar culture medium. Once confluent, the cultures were switched to the air–liquid interface, which corresponds to day zero of age. After a week of culture, the epithelium was fully differentiated.

### 2.2. Histology

Morphology of the epithelium was characterized by histological staining in cross sections. Tissue cultures were washed with phosphate buffered saline without calcium and magnesium (DPBS, PAN-Biotech, Aidenbach, Germany) successively three times before fixation for 30 min with 4% formaldehyde (diluted in DPBS). Fixed tissues were sent in DPBS to Cerba HealthCare (Issy-les-Moulineaux, France) where the cultures were processed and stained with hematoxylin and eosin. Digital images of the stained cultures cross-section were acquired and analyzed with NDP.view2 software (version 2.9.22).

### 2.3. Immunofluorescence (IF)

To characterize the cell types in AlveolAir^TM^, IF was performed using primary antibodies specific to Podoplanin (PDPN, 1:100, Abcam, Cambridge, UK) for ATIs; HTII-280 (1:150, Terrace Biotech, San Francisco, CA, USA) specific to ATIIs; E-cadherin (E-cad, 1:500, Abcam, Cambridge, UK) for adherent junctions; and Zonula Occludens-1 (ZO-1, 1:100, Thermo Fisher Scientific, Waltham, MA, USA), for tight junctions. The secondary antibodies were Alexa Fluor 568 goat anti-mouse IgM antibody, Alexa Fluor 488 goat anti-rabbit antibody, and Alexa Fluor 568 goat anti-mouse IgG antibody (1:500, Invitrogen, Waltham, MA, USA). Cultured tissues were fixed with ice-cold methanol for 15 min at room temperature and blocked for 1 h minimum with 5% fetal calf serum (FCS, PAN Biotech, Aidenbach, Germany). Tissues were incubated for an hour minimum with primary and then secondary antibody before being counterstained with DAPI (1:1000, AppliChem, Chicago, IL, USA) for 10 min. All solutions are diluted in DPBS. Washing with DPBS was carried out in between each incubation. Incubations were performed at room temperature (RT) or overnight at 4 °C. Membranes of the inserts were cut out and mounted with Prolong Antifade Diamond (Invitrogen). Imaging was performed on an LSM800 Airyscan (Zeiss, Oberkochen, Germany).

For the quantification of ATIIs area, IF was performed to label ATIIs and tight junctions using HTII-280 and ZO-1 markers, respectively. Images were captured at 40 X magnification using the LSM800 Airyscan microscope. The surface area of HTII-280-positive cells was measured with ImageJ software (version 1.54f) and expressed as a ratio relative to the total apical area.

### 2.4. Scanning Electron Microscopy (SEM) and Transmission Electron Microscopy (TEM)

The morphology of the epithelium was characterized by scanning electron microscopy (SEM) and transmission electron microscopy (TEM). Tissue cultures were washed with DPBS three times successively before fixation for 30 min with sodium bi-phosphate buffer (0.2 M, pH 7.2) with 2% formaldehyde (*v*/*v*, Sigma-Aldrich, St. Louis, MO, USA) and 2.5% glutaraldehyde (*v*/*v*, Sigma-Aldrich). Fixed tissues were sent in DPBS to Tours University (Tours, France) where the cultures were processed and imaged.

### 2.5. Gene Expression

RNA was extracted from AlveolAir™ tissue with RNeasy Mini kit (Qiagen, Hilden, Germany) and quantified by real-time quantitative polymerase chain reaction (RT-qPCR) with a QuantiTect Probe RT-PCR (Qiagen). Probes against the following genes were purchased from Thermo Fisher Scientific: aquaporin 5 (*AQP5*, hs00387048_m1), caveolin-1 (*CAV-1*, hs00971716_m1), podoplanin (*PDPN*, hs00366766_m1), ATP binding cassette subfamily A member 3 (*ABCA3*, hs00184543_m1), hedgehog interacting protein (*HHIP*, hs01011015_m1), and glyceraldehyde-3-phosphate dehydrogenase (*GAPDH*, hs02758991_g1). Values were normalized with house-keeping gene GAPDH expression using the 2^−∆∆^ method. Gene expression from AlveolAir™ was reported as mean fold-change compared to human lung RNA (AMSBIO, Abingdon, UK).

After viral infection, host gene expression assays were performed using RT-qPCR using the EXPRESS One-Step Superscript™ qRT-PCR Kit (Invitrogen^TM^) with a QuantStudio™ 5 Real-Time PCR System (Applied Biosystems, Waltham, MA, USA) from total RNA extracted from Alveolar cell lysates harvested at 96 h post-infection (hpi). TaqMan probes of the following cytokines and cell death markers of interest were purchased from Thermo Fisher Scientific: *GAPDH* (hs02758991_g1,), Interleukin-6 (*IL-6*, hs00174131_m1, Thermo Fisher Scientific), Interleukin-8 (*CXCL8*, hs00174103_m1), Interferon lambda 1 (*IFNL1*, hs00601677_g1), Interferon gamma-induced protein 10 (*CXCL10*, hs00171042_m1), RANTES (*CCL5*, hs00982282_m1), Interferon beta 1 (*IFNB1*, hs01077958_s1), Caspase 1 (*CASP1*, hs00354836_m1). Relative gene expression was normalized on *GAPDH* expression using the 2^−CT^ method. Data are represented as mean fold-change compared to non-infected condition.

### 2.6. Trans-Epithelial Electrical Resistance (TEER)

TEER was measured as previously described [27,28]. Briefly, after addition of 200 μL of culture medium to the apical compartment of the tissue cultures, resistance was measured across cultures with an EVOMX volt-ohmmeter (World Precision Instruments, Sarasota, USA) for each condition. Resistance values (Ω) were converted to TEER (Ω∙cm^2^) by using the following formula:

TEER (Ω∙cm^2^) = (resistance value (Ω) − 100(Ω)) × 0.33 (cm^2^),

where 100 Ω is the resistance of the membrane and 0.33 cm^2^ is the total surface of the epithelium.

### 2.7. Lucifer Yellow Assay

The Lucifer yellow assay was utilized to evaluate apical-to-basal permeability in AlveolAir™. The apical compartment was filled with 250 µL of 100 µM Lucifer yellow (Sigma-Aldrich), and the basal compartment was filled with 500 µL of Hank’s Balanced Salt Solution (HBSS). Every 30 min for two hours, 200 µL were collected from the basal compartment of AlveolAir™ and replaced with fresh HBSS. After two hours, 200 µL were collected from the apical compartment to quantify the remaining Lucifer yellow. Fluorescence intensity was measured (excitation 360 nm; emission 530 nm) with CytoFluor Series 4000 (Applied Biosystems) from the collected volume to assess the quantity of Lucifer yellow that crossed the epithelium. A standard curve for Lucifer yellow was generated through serial 1:2 dilutions, ranging from 100 µM to 0.1 µM. The limit of detection (LOD) for the fluorescence multi-well plate reader was determined from the standard curve and calculated to be 0.39 µM.

### 2.8. Virus

Influenza H1N1 A/Switzerland/3076/16 clinical specimen was isolated from the nasopharyngeal swab of an anonymized patient, provided from the Geneva University Hospital [29]. Viral stocks were produced in MDCK-NBL-2 cells from an initial stock produced on MucilAir™, provided by Epithelix (Geneva, Switzerland). For amplification, MDCK-NBL-2 cells were seeded in T25 flasks at a density of 1.5 × 10^6^ cells per flask, one day prior to infection, and incubated at 37 °C in a humidified 5% CO_2_ atmosphere until reaching 80–90% confluence. Cells were then infected in MEM (Corning, NY, USA) 15-010-CV) supplemented with 2 mM L-Glutamine (Gibco, Waltham, MA, USA) 25030-123), penicillin (100 U/mL)–streptomycin (100 µg/mL, Gibco, 15140-163), and 1 µg/mL Trypsin Acetylated from bovine pancreas (Sigma-Aldrich, St. Louis, USA) T6763-100MG). Supernatants were collected at 2 days post-infection (dpi), clarified by centrifugation at 700 g for 5 min, aliquoted, and stored at −80 °C. Viral titers were determined by tissue culture infectious dose 50% (TCID_50_/mL) in MDCK-NBL-2.

The SARS-CoV-2 strain B.1.617.2 (Delta) used in this study was sequenced and deposited in the GISAID EpiCoV database under the reference hCoV-19/France/ARA-VIRPATH-01/2020 (accession ID EPI_ISL_7360393). For amplification, Vero E6 cells were seeded in T175 flasks and infected at 80–90% confluence in DMEM with 4.5 g/L glucose (Corning) supplemented with 2 mM L-Glutamine (Gibco), penicillin (100 U/mL)–streptomycin (100 µg/mL, Gibco), and 2% FBS (Dutscher, Bernolsheim, France). Supernatants were collected at 4 dpi, clarified by centrifugation at 3000 rpm for 9 min, aliquoted, and stored at −80 °C. Viral titers were determined by TCID_50_/mL in Vero E6 cells, as previously described [30].

### 2.9. Viral Infection and Treatment in AlveolAir™

Apical side of AlveolAir^TM^ were gently washed twice with warm Opti-MEM^®^ medium (Gibco) and then inoculated directly with 150 µL of Opti-MEM^®^ medium for non-infected condition (n = 11) or with dilution of virus in 150 µL of Opti-MEM^®^ medium, at a MOI of 10^−1^ for SARS-CoV-2 (n = 8) or MOI 1.6 × 10^−3^ for Influenza H1N1 strain (n = 10). Apical inocula were washed away after 1 h of incubation and AlveolAir^TM^ cultures were then incubated at 37 °C in a humidified 5% CO_2_ atmosphere during the time-course of infection. Infected AlveolAir^TM^ epithelium was treated or not, depending on the virus used, by renewal of basal medium by antiviral molecules dilution (n = 3/treatment) in 700 µL of AlveolAir^TM^ culture medium (Epithelix, Geneva, Switzerland). For Delta infection, Remdesivir (5 µM, MedChemExpress, Princeton, NJ, USA) treatment was administered at 1 hpi, 24 hpi, 48 hpi, and 72 hpi. For Influenza infection, Baloxavir (10 nM, MedChem) treatment was administered at 0 hpi, 24 hpi, 48 hpi, and 72 hpi. As control for inflammation, non-infected AlveolAir^TM^ (n = 3) cultures were treated with cytomix, which is a mixture of Lipopolysaccharide (LPS, 0.2 mg/mL, Sigma-Aldrich), Tumor necrosis factor alpha (TNF-α, 0.5 µg/mL, BioLegend, San Diego, CA, USA), and 1% FCS (PAN Biotech) at 0 hpi, 24 hpi, 48 hpi, and 72 hpi.

Apical washes were realized at 24 hpi, 48 hpi, 72 hpi, and 96 hpi for virus sampling and quantification by TCID_50_ or RT-qPCR. Basolateral medium was collected at 24 hpi, 48 hpi, 72 hpi, and 96 hpi for cytokines dosage. Alveolar cells and endothelial cells were harvested separately at 96 hpi in RLT buffer (Qiagen) to extract total RNA from cell lysates for subsequent RT-qPCR quantification.

### 2.10. Virus Quantification

MDCK-NBL-2 cells (ATCC, Manassas, VA, USA) were maintained in Minimum Essential Medium with Earle’s salts (MEM, Corning, New York, NY, USA) supplemented with 2 mM L-Glutamine (Gibco, Waltham, MA, USA), penicillin (100 U/mL)–streptomycin (100 µg/mL, Gibco), and 10% fetal bovine serum (FBS, Dutscher, Bernolsheim, France). Vero E6 (ATCC) were maintained in Dulbecco’s Modified Eagle’s Medium (DMEM, Corning) with 4.5 g/L glucose supplemented with 2 mM L-Glutamine (Gibco), penicillin (100 U/mL)–streptomycin (100 µg/mL, Gibco), and 5% FBS (Dutscher).

Viruses from apical wash samples were titrated by TCID_50_/mL in Vero E6 cells for SARS-CoV-2 strain or MDCK NBL-2 cells for Influenza H1N1 strain, using the Reed and Muench statistical method, as previously described [31]. In parallel, RNA was extracted from the apical washes with the QIAamp^®^ Viral RNA kit (Qiagen) or from cell lysate with RNeasy^®^ Mini kit (Qiagen). Absolute quantification of viral genome was performed by quantitative RT-qPCR with EXPRESS One-Step Superscript^TM^ qRT-PCR Kit (Invitrogen^TM^) and QuantStudio™ 5 Real Time PCR System (Applied Biosystems) using primers and TaqMan probe targeting the SARS-CoV2 ORF1b-nsp14 (forward primer HKU-ORF1b-nsp14F: 5′-TGGGGYTTTACRGGTAACCT-3′; reverse primer HKU-ORF1b-nsp14R: 5′ AACRCGCTTAACAAAGCACTC-3′; probe HKU-ORF1b-nsp141P: 5′-FAM TAGTTGTGATGCWATCATGACTAG-TAMRA-3′) or the Influenza M gene (forward primer 5′-CTTCTAACCGAGGTCGAAACGTA-3′; reverse primer 5′-GGTGACAGGATTGGTCTTGTCTTTA-3′; probe 5′-FAM TCAGGCCCCCTCAAAGCCGAG BHQ-3′). A calibration curve was made by amplification of in vitro transcribed nsp14 RNA for SARS-CoV2 or M RNA for Influenza H1N1.

### 2.11. Enzyme-Linked Immunosorbent Assay (ELISA)

Basal media were collected after viral infection at 24 hpi, 48 hpi, 72 hpi, and 96 hpi and centrifugated at 1500 rpm for 10 min. A volume of 190 µL of basal medium was then mixed with 10 µL of a 20X TnBP-Triton-X100 solution, freshly prepared as follows: 740 µL DPBS (Gibco), 60 µL TnBP (EMPROVE^®^ EXPERT Ph Eur, Merck, Rahway, NJ, USA) and 200 µL Triton X-100 (Sigma-Aldrich). The mixture was then incubated at room temperature for 2 h and frozen at −80 °C. IL-8 (BD Biosciences, Franklin Lakes, USA), IL-6 (BD Biosciences), and RANTES (BD Biosciences) were quantified by ELISA in basal medium according to manufacturer’s instructions.

### 2.12. Statistics

Data were expressed as mean ± standard error of the mean. For statistical analysis, two-way ANOVA was performed with multiple comparison tests using GraphPad Prism software (GraphPad Prism, version 6.01, San Diego, CA, USA) (* *p* < 0.05, ** *p* < 0.01, *** *p* < 0.001, **** *p* < 0.0001).

## 3. Results

### 3.1. Morphological Characterization of AlveolAir™: The Presence of ATIs and ATIIs

**Figure 1:**
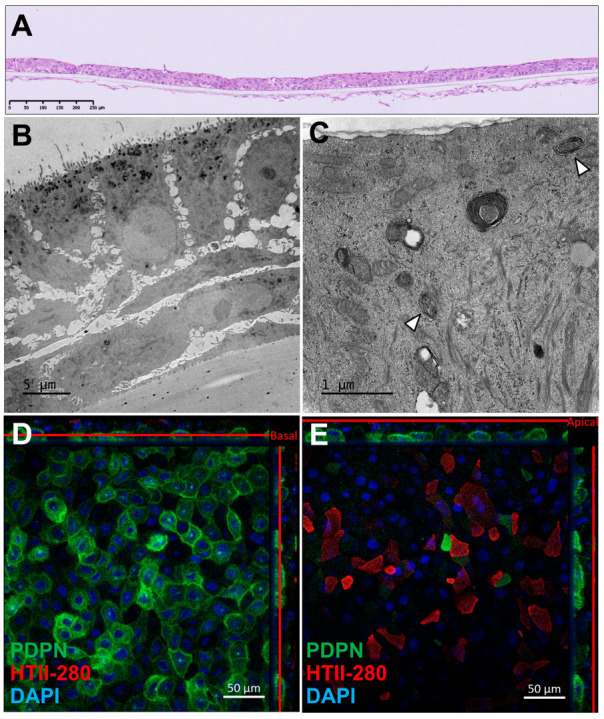
Morphological characterization of AlveolAir™. (**A**) Representative cross-section of AlveolAir™, on day 11, stained with hematoxylin and eosin. (**B**,**C**) Transmission electron microscopy (TEM) images of AlveolAir™ cross-sections on days 20 and 18, respectively. (**C**) Arrowheads show lamellar bodies. (**D**,**E**) Representative confocal immunofluorescence staining on day 18 of pneumocytes type 2 (ATII) protein HTII-280 (red) and pneumocyte type 1 (ATI) protein Podoplanin (PDPN, green) and nuclei (DAPI, blue). Acquisition of representative images was performed with a confocal inverted microscope Zeiss Confocal LSM 800 (Zeiss, Oberkochen, Germany). The image is displayed in the X-Y projection, accompanied by orthogonal views positioned above and to the right of the main image. A red bar is included to indicate the Z position corresponding to the X-Y projection.

Immunohistochemical analysis of AlveolAir™, stained with hematoxylin and eosin, revealed a multilayered epithelial and endothelial tissue, respectively, on the apical and basolateral side of the insert. The epithelial tissue consisted of three to four cell layers and measured approximately 30 µm in thickness (Figure 1A). Transmission electron microscopy (TEM) revealed a thinner, three-layered epithelial tissue, with a thickness of around 20 µm (Figure 1B). The basal cells adhering to the plastic membrane exhibited an elongated morphology, while the cells in the upper layers displayed a cuboidal shape. TEM imaging at higher magnification (Figure 1C) identified ATII, as evidenced by the presence of lamellar bodies (arrows), a hallmark of ATII cells. To further characterize the cellular composition, immunofluorescence staining was performed on day 18 to assess the expression of podoplanin (PDPN), a marker of ATI, and HTII-280, a specific marker for ATIIs (Figure 1D,E). These panels, representing different Z-positions of the same tissue, revealed a basal layer covered with rounded PDPN-positive cells, while the apical layer predominantly composed of angular HTII-280-positive cells. Additionally, a subset of PDPN-positive cells exhibited a morphology similar to HTII-280-positive cells. Finally, DAPI staining evidenced a two-layered tissue. Despite batch-to-batch variability in tissue thickness, the localization and expression of ATI and ATII markers were consistently observed, indicating the robust generation of alveolar-like tissue in AlveolAir™.

### 3.2. Molecular Characterization of AlveolAir™: An Increasing Number of ATIIs over Time

**Figure 2:**
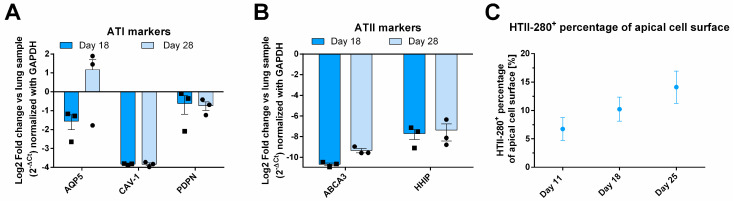
Molecular characterization of AlveolAir™. The relative quantification of gene expression from one sample of human lung parenchyma was correlated to gene expression in AlveolAir™. (**A**,**B**) Epithelial tissues were lysed at days 18 and 28, and from the purified RNA, the genes of interest were measured by RT-qPCR (n = 3, mean ± SEM). Relative gene expression was normalized by GAPDH house-keeping gene and presented as mean fold-change compared to human lung parenchyma. (**A**) ATI markers AQP5, CAV-1, and PDPN gene expression were assessed. (**B**) ATII markers ABCA3 and HHIP gene expression were evaluated. (**C**) HTII-280-positive cells were visualized by IF and their surface quantified and expressed as a percentage of apical cell surface (n = 3, mean ± SEM).

To further investigate the cellular composition of the epithelial layer, RT-qPCR analysis was conducted on tissue samples from days 18 and 28, with results presented as fold change relative to primary lung RNA (Figure 2A,B). The expression of ATI genes, CAV-1 and PDPN, remained stable over time. PDPN expression in AlveolAir™ was approximately twofold lower compared to that in lung tissue, while CAV-1 expression was markedly reduced, showing a consistent 16-fold decrease relative to lung levels. In contrast, the expression of the ATI marker AQP5 exhibited temporal variability between days 18 and 28. However, overall, its expression in AlveolAir™ was comparable to that observed in lung tissue (Figure 2A). Additionally, to identify ATIIs, ATP-binding cassette subfamily A member 3 (ABCA3) and hedgehog interacting protein (HHIP) were analyzed (Figure 2B). Both markers exhibited stable expression over time in AlveolAir™, although their expression levels were considerably lower than those observed in lung RNA (Figure 2B). The reduced expression of pneumocyte markers in AlveolAir™ relative to lung samples revealed some notable quantitative differences between in vitro and in vivo alveoli. However, qualitatively, AlveolAir™ shares the key alveolar characteristics of the lung over time. The IF analysis of ATII biomarker HTII-280 showed that between days 11 to 25, HTII-280-positive surface increased from 5 to 15% of the total apical surface (Figure 2C). These results indicate that AlveolAir™ recapitulates in vivo surface proportion of ATIIs at earlier time points.

### 3.3. AlveolAir™ Exhibits a Long-Term Barrier Function

**Figure 3:**
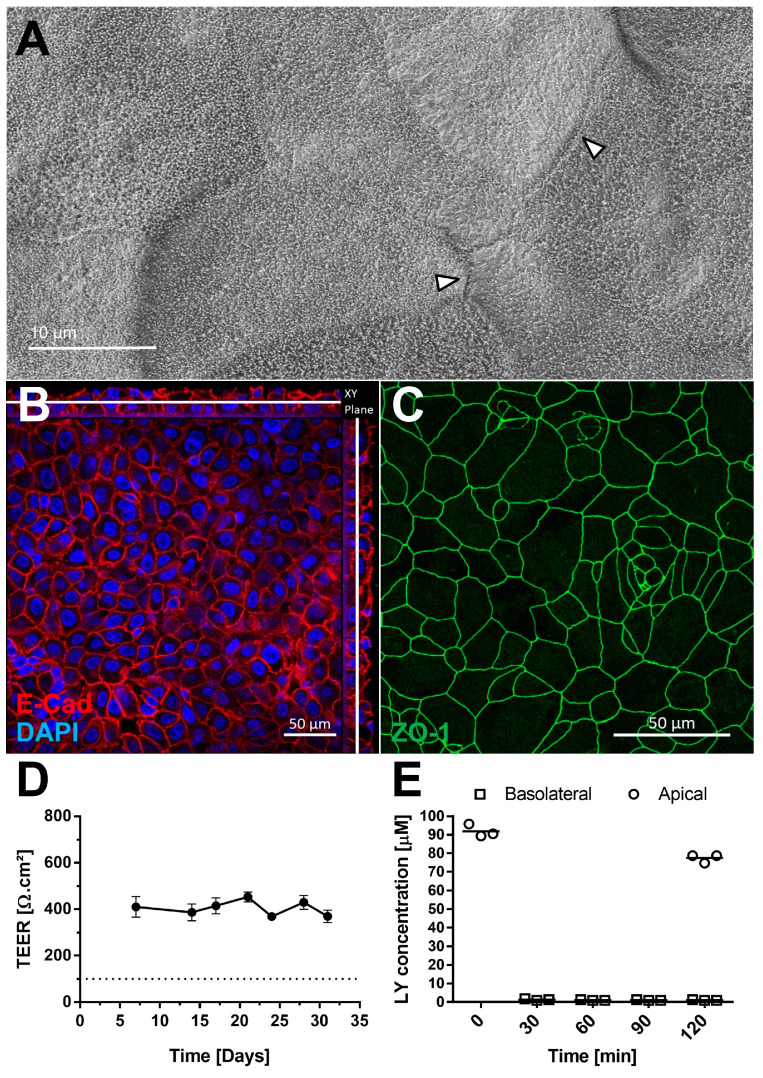
AlveolAir™ barrier functions. (**A**) Scanning electron microscopy (SEM) images of AlveolAir™ apical surface on day 18, arrowheads indicating tight junctions between cells. (**B**,**C**) Acquisition of representative images areas was performed with a confocal inverted microscope Zeiss Confocal LSM 800. (**B**) Immunofluorescence staining of adherent junctions (E-cadherin, red) and nucleus (DAPI, blue). The image is displayed in the X-Y projection, accompanied by orthogonal views positioned above and to the right of the main image. A white bar is included to indicate the Z position corresponding to the X-Y projection. (**C**) Immunofluorescence staining of tight junctions (ZO-1, green). (**D**) Kinetic analysis of transepithelial electrical resistance (TEER) (n = 7, mean ± SEM). (**E**) Permeability of AlveolAir™ to Lucifer yellow (n = 3, mean ± SEM).

The presence of tight junctions was examined by scanning electron microscopy (Figure 3A) and confirmed by IF using an antibody against Zonula Occludens-1 (ZO-1) (Figure 3C). Cell sizes ranged from 10 to 30 µm (Figure 3C). Adherent junctions were detected via E-cadherin staining, with orthogonal Z-stack views showing their presence throughout the tissue (Figure 3B). AlveolAir™ cultures maintained a stable transepithelial electrical resistance (TEER) value of approximately 400 Ω∙cm^2^ (n = 7) up to day 31 (Figure 3D), indicating long-term barrier stability. Permeability testing with Lucifer yellow demonstrated minimal diffusion from the apical to the basolateral compartment 2 h after addition, thereby confirming barrier integrity of AlveolAir™ (Figure 3E). These results collectively suggest that AlveolAir™ forms a stable and functional barrier, suitable for long-term toxicity and permeability assays.

### 3.4. H1N1 Replication Alters Integrity of AlveolAir™ and Induces a Strong Inflammatory Response in Epithelial Cells

**Figure 4:**
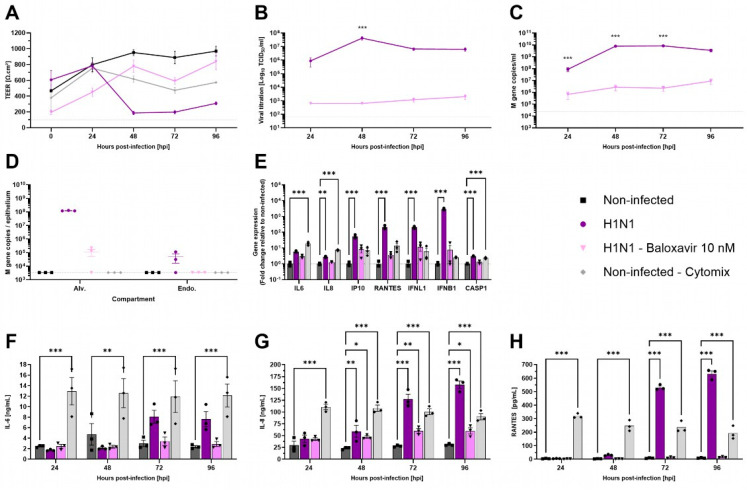
Characterization of H1N1 infection in AlveolAir™ model. AlveolAir™ epithelium was infected with H1N1 A/Switzerland/3076/2016 strain at a MOI of 1.6 × 10^−4^ (n = 6) or mock-inoculated with Opti-MEM^®^ medium (non-infected, NI, n = 3). Baloxavir treatment (10 nM) was administered in the basal medium of infected AlveolAir™ at 0 h post-infection (hpi), 24 hpi, 48 hpi, and 72 hpi (n = 3). As control for inflammation, non-infected AlveolAir™ were exposed to a cytomix mixture of LPS, TNF-α, and FCS in basal medium at different time points (n = 3). (**A**) TEER was measured daily from 0 hpi to 96 hpi and expressed as Ω∙cm^2^. (**B**) Infectious titers were measured in MDCK cells from apical washes harvested daily by TCID50 assays. (**C**,**D**) Virus replication was measured by RT-qPCR quantification of viral M gene from (**C**) apical wash samples harvested daily after infection or (**D**) cellular lysates of alveolar cells (Alv.) or endothelial cells (Endo.) harvested at 96 hpi. Absolute M gene quantification was calculated using a standard curve. (**E**) Host genes (IL-6, IL-8, CXCL10, RANTES, IFNL1, and IFNB1) expression after infection and/or treatment were measured by RT-qPCR from cellular lysates of alveolar cells at 96 hpi. Relative gene expression was normalized on GAPDH expression and represented as mean fold-change compared to NI condition. (**F**,**H**) IL-6 (**F**), IL-8 (**G**), and RANTES (**H**) cytokines were quantified by ELISA assays from basal samples. Results are represented as mean ± SEM. Statistical significance was calculated by two-way ANOVA in comparison to H1N1 group (**B**,**C**) or NI group (**F**–**H**) or by one-way ANOVA in comparison to NI group ((**D**,**E**); * *p* < 0.05, ** *p* < 0.01, *** *p* < 0.001).

Respiratory viruses exhibit specific tropism within the respiratory tract. Influenza viruses are known to replicate in ATI and ATII cells [32]. To evaluate the functionality of AlveolAir™, cultures were infected with H1N1 virus, either in the presence or absence of antiviral treatment using 10 nM Baloxavir. TEER measurements demonstrated a marked reduction from 48 h post-infection (hpi) to 96 hpi in H1N1-infected cultures (Figure 4A). This reduction was accompanied by significant epithelial barrier disruption, as evidenced by immunostaining of tissue sections at 96 hpi (Figure S1). In contrast to non-treated cultures, Baloxavir treatment maintained TEER above 400 Ω∙cm^2^, similar to mock-infected AlveolAir™ controls (Figure 4A).

In accordance with these results, infectious viruses released at the apical surface peaked at 48 hpi, reaching 4.21 × 10^7^ TCID_50_/mL, and remained elevated through 96 hpi (Figure 4B). Viral genome quantification corroborated these results, revealing 3.49 × 10^9^ M gene copies in apical washes and 1.22 × 10^8^ M gene copies within alveolar cells at 96 hpi (Figure 4C,D). In contrast, Baloxavir treatment significantly reduced viral replication, with apical titers remaining below 2.01 × 10^3^ TCID_50_/mL at 96 hpi. Viral genome copies in apical washes were markedly lower in Baloxavir-treated cultures compared to untreated conditions (Figure 4B,C). Furthermore, concomitant infection and Baloxavir treatment effectively limited alveolar cells infection as evidenced by the low levels of M gene detected in epithelial cells harvested at 96 hpi (Figure 4D).

Then, pro-inflammatory genes expression in alveolar cells was analyzed at 96 hpi. Compared to non-infected AlveolAir™, significant upregulation of IL-8, IP-10, RANTES, IFNL1, IFNB1, and CASP1 gene expression was measured in H1N1 infected cultures, although the inflammatory profile differed from that observed with an inflammatory cocktail (Cytomix, Figure 4E). These results were corroborated by quantification of IL-6, IL-8, and RANTES secretion into the basal medium over the infection time-course. In comparison to non-infected culture, H1N1 infection induced significantly the secretion of IL-8 (158.04 ng/mL), IL-6 (7.63 ng/mL), and RANTES (631.93 pg/mL) at 96 hpi (Figure 4F–H). These findings suggest that AlveolAir™ serves as a robust platform for investigating the mechanisms of H1N1 infection.

### 3.5. SARS-CoV-2 Delta Variant Infects AlveolAir™ with No Impact on Epithelium Integrity

**Figure 5:**
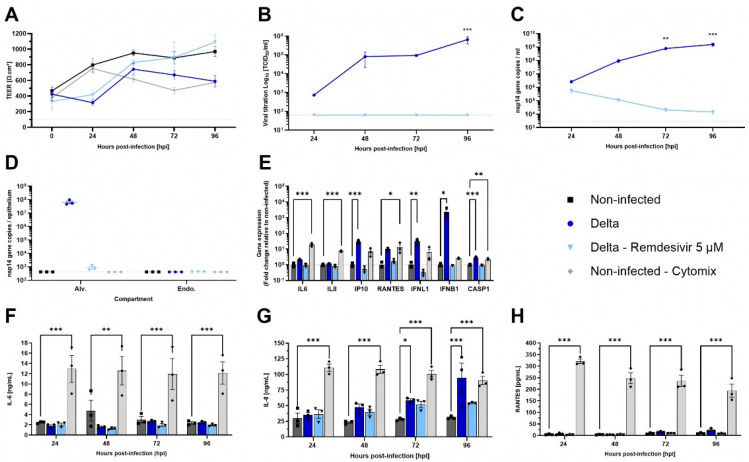
Characterization of SARS-CoV-2 infection in AlveolAir^TM^ model. AlveolAir™ cultures (n = 3/condition) were infected with SARS-CoV-2 Delta strain (B.1.617.2) at a MOI of 0.1 (n = 5) or mock-inoculated with Opti-MEM^®^ medium (non-infected, NI, n = 3). Remdesivir treatment (5 µM) was administered in the basal medium 1 h post-infection (hpi), 24 hpi, 48 hpi, and 72 hpi (n = 3). As control for inflammation, non-infected AlveolAir™ cultures were exposed to a cytomix mixture of LPS, TNF-α, and FCS in basal medium at different time points (n = 3). (**A**) TEER was measured daily from 0 hpi to 96 hpi and expressed as Ω∙cm^2^. (**B**) Infectious titers were measured in Vero E6 cells from apical washes harvested daily by TCID_50_ assays. (**C**,**D**) Virus replication was measured by RT-qPCR quantification of viral nsp14 gene from (**C**) apical wash samples harvested daily after infection or (**D**) cellular lysates of alveolar cells (Alv.) or endothelial cells (Endo.) harvested at 96 hpi. Absolute nsp14 gene quantification was calculated using a standard curve. (**E**) Host genes (IL-6, IL-8, CXCL10, RANTES, IFNL1, and IFNB1) expression after infection and/or treatment were measured by RT-qPCR from cellular lysates of alveolar cells at 96 hpi. Relative gene expression was normalized on GAPDH expression and represented as mean fold-change compared to NI condition. (**F**–**H**) IL-6 (**F**), IL-8 (**G**), and RANTES (**H**) cytokines were quantified by ELISA assays from basal samples. Results are represented as mean ± SEM. Statistical significance was calculated by two-way ANOVA in comparison to the H1N1 group (**B**,**C**) or NI group (F-H) or by one-way ANOVA in comparison to the NI group ((**D**,**E**); * *p* < 0.05, ** *p* < 0.01, *** *p* < 0.001).

Besides influenza viruses, SARS-CoV-2 has been shown to have a lesser impact on the alveolar region in vivo [33]. To evaluate the permissiveness of AlveolAir™ to SARS-CoV-2 and the antiviral efficacy of 5 µM of Remdesivir, cultures were infected with Delta strain B.1.617.2. Unlike H1N1 infection, Delta infection resulted in only a slight decrease in TEER at 48 hpi (Figure 5A). Moreover, at 96 hpi, Delta replication was confined to the upper cell monolayer of AlveolAir™, as demonstrated by immunostaining (Figure S2). From 24 to 96 hpi, the virus particles’ release and replication increased, peaking at 6.52 × 10^5^ TCID_50_/mL and 1.55 × 10^9^ and 6.71 × 10^7^ nsp14 gene copies at 96 hpi, respectively, in apical washes or inside alveolar cells (Figure 5B–D). Compared to non-treated condition, infected AlveolAir™ culture treated with Remdesivir prevented completely Delta replication, represented by no infectious virus released and a progressive clearance of virus genome through the kinetics (Figure 5B–D). Accordingly, Remdesivir-treated cultures showed similar TEER values as non-infected AlveolAir™ (Figure 5A).

Then, pro-inflammatory genes expression in the epithelial compartment was assessed at 96 hpi, comparing infected or treated cultures to non-infected AlveolAir™. IP-10, IFNL1, IFNB1, and CASP1 gene expressions were significantly induced by Delta infection, whereas Remdesivir treatment prevented it efficiently with levels similar to non-infected cultures (Figure 5E). Although cytokine dosage confirmed that Delta infection did not induce secretion of IL-6 or RANTES throughout the time-course of infection, it revealed that it induced significant secretion of IL-8 at 72 and 96 hpi (Figure 5F–H). These results highlight a differential sensitivity of AlveolAir™ to SARS-CoV-2 compared to H1N1.

## 4. Discussion

The aim of this study was to establish a versatile model suitable for various applications, including toxicity testing and studies of respiratory viral infections, characterized by ease of handling, compatibility with long-term use, and medium-throughput capabilities. To achieve this, we developed a co-culture model comprising primary epithelial and endothelial cells exhibiting barrier functionality, which was effectively infected by H1N1 and SARS-CoV-2 viruses. We demonstrated the presence of primary ATIs and ATIIs in AlveolAir™ up to one month (Figure 1C–E and Figure 2), highlighting a key advantage of this system. Even though previous studies have also reported the presence of primary ATIs and ATIIs, the persistence of ATIIs at the air–liquid interface (ALI) culture over extended time points is a novel finding [22,34,35,36]. Notably, gene expression levels of pneumocyte markers in AlveolAir™ were considerably lower than in lung samples, suggesting key differences between the in vitro system and the in vivo alveoli. This discrepancy may stem from the spatial and geometrical differences between in vitro and in vivo systems, as well as the difference in cellular compositions. Refining culture conditions, such as optimizing medium composition and new insert design, could enhance pneumocyte maturation and improve differentiation. Co-culture with other cell types such as alveolar macrophages may also be an option.

Current models are typically maintained at the ALI for short durations ranging from 3 to 14 days, limiting their use for long-term assays [34,35,37,38]. In contrast, AlveolAir™ demonstrated stable ATII gene expression over time, accompanied by a trend toward increased number of ATIIs counted on the apical surface during long-term culture (Figure 2). This finding diverges from previous reports, which often describe a tendency of ATIIs’ differentiation into ATIs, leading to a decrease in ATIIs numbers over time [34]. Yang et al. highlighted the influence of culture conditions on ATIIs maintenance by demonstrating that transitioning epithelial cultures from submerged to ALI conditions resulted in an initial decrease in ATIIs during the first 7 days at the ALI, followed by stabilization between days 7 and 14. This underlines the importance of optimized culture conditions in developing physiologically relevant alveolar model.

In our study, AlveolAir™ displayed adherent and tight junctions, which are essential for applications in toxicity and drug transport assays (Figure 3). Beyond serving as a physical barrier, pneumocytes also act as immunomodulators by releasing chemokines and cytokines. To evaluate this immunomodulatory capacity, we measured IL-6 and IL-8 secretion in AlveolAir™, following stimulation with pro-inflammatory mediators (Figure S2). AlveolAir™ exhibited distinct responses to poly(I:C), LPS, and TNF-α. Notably, while Mitchell et al. reported higher IL-6 levels in their co-culture model, their stimulations utilized LPS and TNF-α concentrations that were 5- and 10-fold higher, respectively, than those applied in our study. Additionally, AlveolAir™ secreted higher levels of IL-8, suggesting increased sensitivity compared to their model [39]. Poly(I:C), a synthetic double-stranded RNA used to mimic viral infection, was the most potent stimulator, significantly upregulating IL-6 and IL-8 expression and release. This robust immune response supports the suitability of AlveolAir™ for studying viral infections.

The presence of ATIIs is critical for studying viral infections, as both H1N1 and SARS-CoV-2 predominantly infect these cells in the alveolus [33,40,41]. Observations from AlveolAir™ suggested infection of ATIIs by both viruses (Figure S2). However, the colocalization of viral proteins with HTII-280 was limited, suggesting that viruses could also infect other types of cells. Further analysis using additional ATII/ATI markers is needed to better identify viral tropism in AlveolAir™.

Interestingly, the responses to H1N1 and SARS-CoV-2 differed, highlighting distinct pathophysiological mechanisms and the versatility of AlveolAir™ in modeling these infections. For H1N1, viral replication was observed exclusively in epithelial cells, with no replication detected in endothelial cells. The presence of H1N1 M gene copies in two out of three replicates suggests potential contamination or leaking from apical to basal compartment due to loss of tissue integrity rather than genuine infection of endothelial cells. H1N1 infection had a pronounced impact on tissue integrity, evidenced by a significant decrease in TEER and tissue loss, as observed in immunohistochemical cross-sections (Figure 4A and Figure S1A). These findings are consistent with previous reports in the literature [42]. Gene expression analysis revealed upregulation of host genes associated with inflammation and immune cell activation, similar to findings by Yu et al. [32]. H1N1 induced a strong secretion of cytokines, particularly at later time points, an effect that was mitigated by the antiviral compound Baloxavir. These results underscore the capacity of AlveolAir™ to model the inflammatory responses associated with H1N1 infection and evaluate the efficacy of antiviral treatments.

In the case of SARS-CoV-2, infection was also confirmed in ATIIs (Figure S2B), consistent with reports in the literature [36]. Notably, endothelial cells were not infected, corroborating findings by Wang et al., who detected minimal SARS-CoV-2 nucleocapsid protein in endothelial cells within the epithelial-endothelial co-cultures. This is likely due to the low expression of the ACE2 receptor in endothelial cells compared to epithelial cells. Wang et al. observed a broad modulation of the endothelial cell proteome, which they speculated was driven by cytokines secreted by epithelial cells, although these cytokines were not quantified in their study [43]. Consistent with this hypothesis, we detected a substantial increase in the secretion of the inflammatory cytokine IL-8 at later time points. Additionally, we observed significant upregulation of interferon-related genes expression, further supporting the role of epithelial cell-derived cytokines in influencing endothelial cell responses.

These distinct viral behaviors also extend to replication kinetics. H1N1 exhibits similar replication kinetics in nasal epithelial cells (MucilAir™) and alveolar epithelial cells. According to Lamballerie et al., replication in nasal epithelial cells reaches a plateau at 24 h, quantified at 10^8^ TCID_50_/mL [44]. In AlveolAir™, however, the plateau occurs later, at 48 h, with a titer of 4.21 × 10^7^ TCID_50_/mL.

In contrast, SARS-CoV-2 shows a progressive decline in peak replication along the respiratory tract. Pizzorno et al. reported peak viral titers of 10^7^ TCID_50_/mL in nasal epithelial cells (MucilAir-Nasal) and 10^6^ TCID_50_/mL in bronchial epithelial cells (MucilAir-Bronchial) [30]. In AlveolAir™, replication peaked at 6.5 × 10^5^ TCID_50_/mL, suggesting lower tissue permissiveness in deeper lung regions, likely due to the decreasing density of ACE2 receptors [45].

These viral replication patterns are in line with interferon responses. In nasal epithelial cells, IFNB1 expression remained 50-fold upregulated from 48 h post-infection (hpi). In bronchial epithelial cells, IFNB1 peaked at 48 hpi before returning to near-baseline by 96 hpi [30]. In alveolar epithelial cells, however, IFNB1 expression was ~10³-fold upregulated at 96 hpi compared to uninfected controls. These findings emphasize distinct influenza and SARS-CoV-2 replication kinetics across respiratory models. In this context, IFN type I and other IFN expressions would be investigated further considering infection kinetics in several physiologically and pharmacologically relevant models.

Together, these findings reinforce the physiological relevance of AlveolAir™ as a model for respiratory viral infections. The efficacy of Baloxavir against H1N1 and Remdesivir against SARS-CoV-2 in reducing viral replication demonstrates the suitability for evaluating antiviral treatments. As a co-culture of primary epithelial and endothelial cells forming a functional barrier, AlveolAir™ replicates viral response patterns consistent with those reported in the literature, offering a robust system for studying host–pathogen interactions and assessing therapeutic interventions.

To closely mimic in vivo structure, physiological models should be of a single-cell layer, with intercalated ATI and ATII cells. While Tanabe et al. achieved a monolayer culture of epithelial cells by day 7, their tissue required frequent passaging, which suggested a risk of becoming multilayered if not properly maintained [35]. Similarly, Carius et al. developed a cell line from a single-cell clone and achieved a monolayer on day 14 [22]. In contrast, AlveolAir™ exhibited some batch-to-batch variability in tissue thickness, ranging from two to four cell layers (Figure 1A,B). Despite this multilayered structure, the functional assessments of AlveolAir™ yielded results consistent with those reported in the literature, indicating that the tissue thickness did not adversely affect the model’s performance or physiological relevance for viral infection studies and drug development.

AlveolAir™ currently exhibits specific constraints, including a functional lifespan of approximately 30 days and an uneven distribution of ATI and ATII cells on the apical layer. To overcome these challenges, future optimization efforts could focus on engineering a uniform monolayer of cells to more accurately mimic the structural organization of in vivo alveoli. Additionally, further advancements could involve the evaluation of antiviral responses using diseased epithelial cells derived from donor stratification based on conditions such as asthma or COPD.

Future research could also explore the comparative effects of systemic versus inhaled drug administration, the impact of infections over extended periods, and the development of immunomodulators and antivirals. Furthermore, investigating AlveolAir™’s sensitivity to environmental stressors and integrating it with immune cell co-cultures would significantly expand its scope. These enhancements would increase the model’s relevance and utility for studying respiratory biology, virology, and toxicology.

## 5. Conclusions

In conclusion, this study demonstrated that AlveolAir™ possesses significant advantages, including the sustained presence of ATIIs over extended periods and a reliable barrier function. Furthermore, it has proven relevant for modeling respiratory infections caused by H1N1 and SARS-CoV-2, effectively capturing key differences in their pathophysiological effects and immune responses. These findings establish AlveolAir™ as a robust platform for drug screening against Influenza and SARS-CoV-2 and underscore its value as a versatile tool for investigating host–pathogen interactions and inflammatory processes.

## Data Availability

Data are contained within the article and Supplementary Materials.

## Supplementary Materials

The following supporting information can be downloaded at: https://www.mdpi.com/article/10.3390/microorganisms13030572/s1, Figure S1: Morphological characterization of SARS-CoV-2 and H1N1 infection in AlveolAir^TM^ model.; Figure S2: Daily repeated apical exposure to pro-inflammatory compounds on AlveolAir™.
