# Supplementary figures and images for: A Novel In Vitro Primary Human Alveolar Model (AlveolAir™) for H1N1 and SARS-CoV-2 Infection and Antiviral Screening
Source: Microorganisms. 2025 Mar 3;13(3):572. doi: 10.3390/microorganisms13030572 (PMC11944821; doi:10.3390/microorganisms13030572)

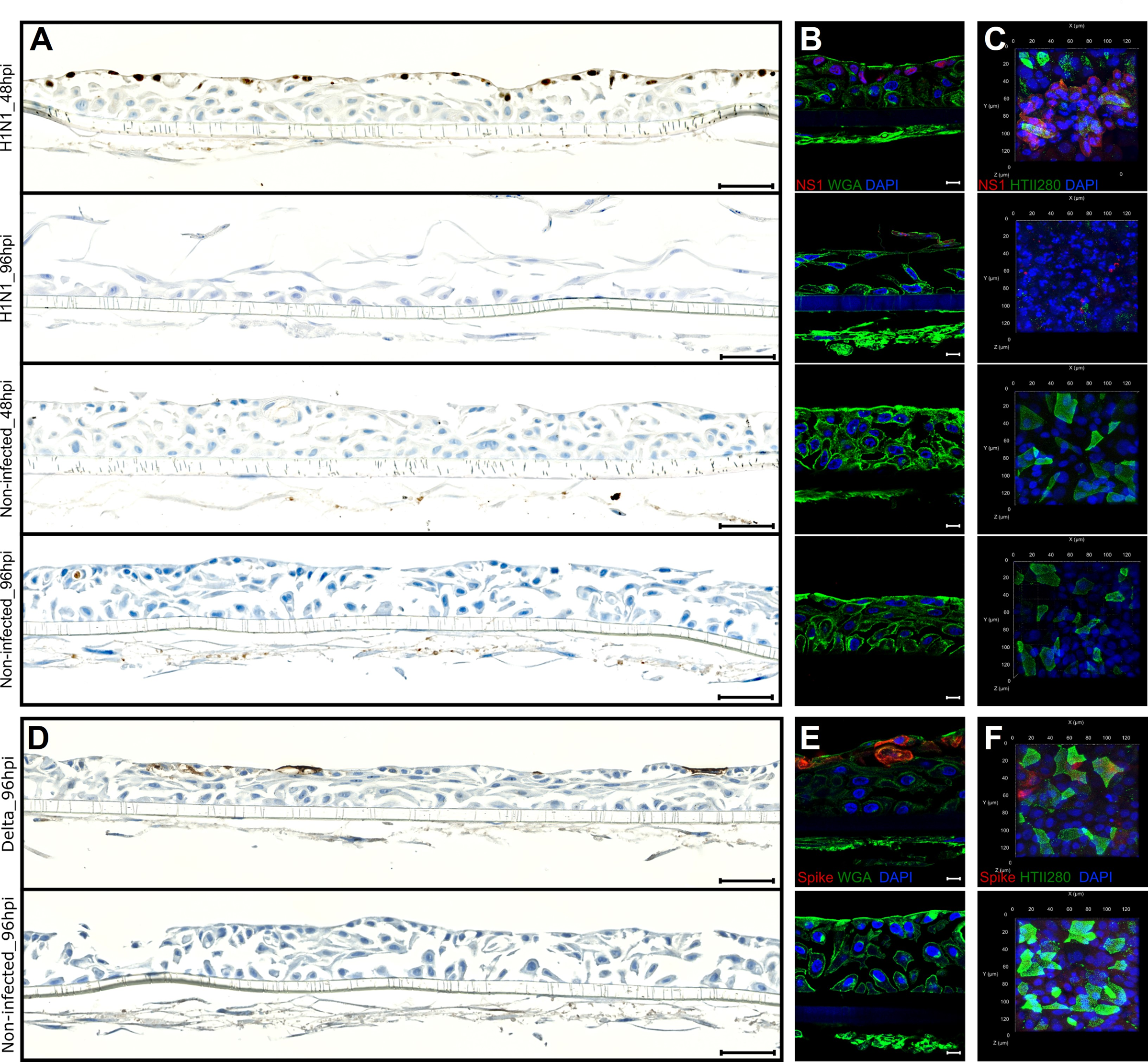

Supplement: Supplementary file 1 [file microorganisms-13-00572-s001.zip › Supplementary figure s1.png]

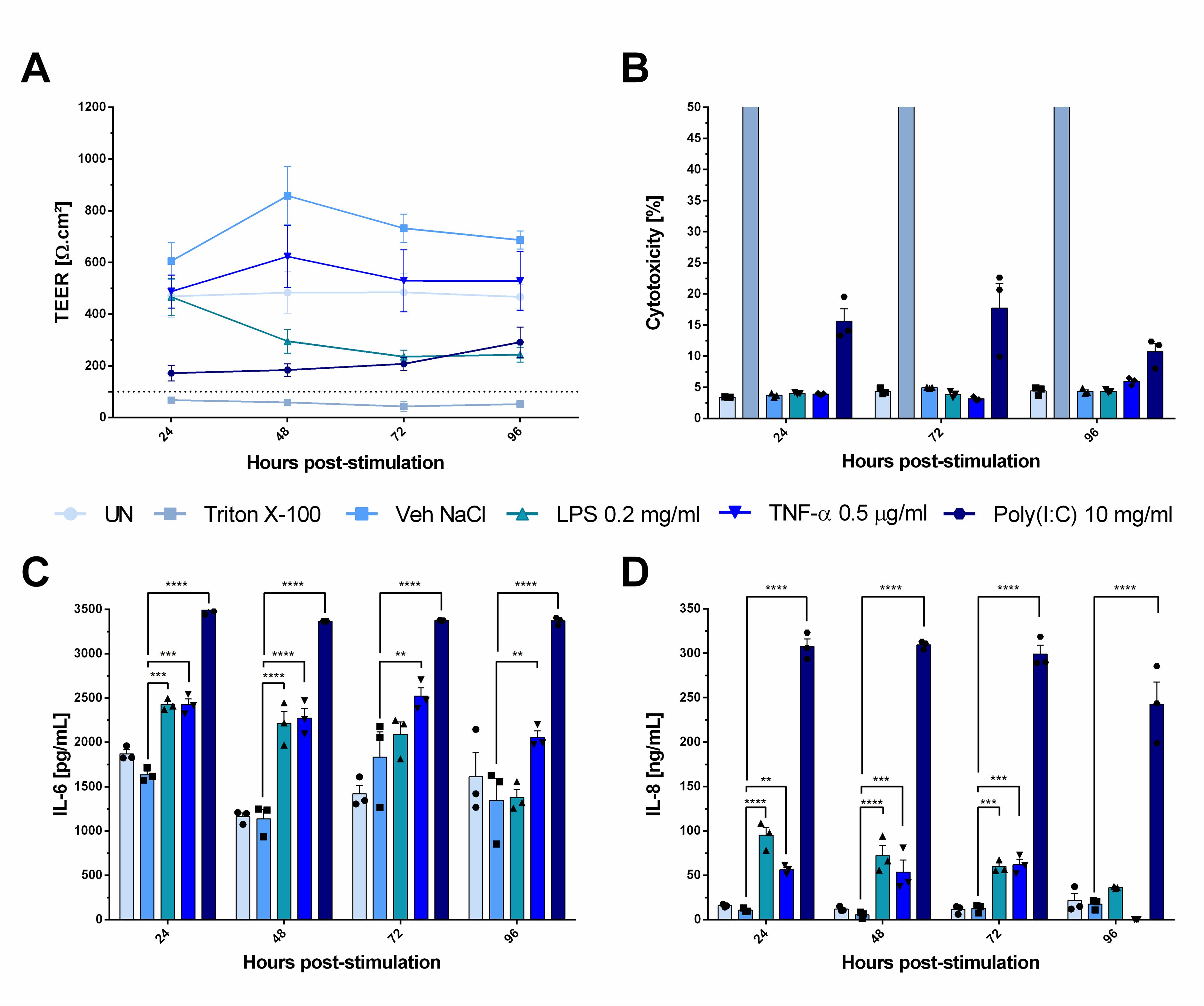

Supplement: Supplementary file 1 [file microorganisms-13-00572-s001.zip › Supplementary figure s2.png]
